# Behavioral and transcriptome alterations in male and female mice with postnatal deletion of TrkB in dorsal striatal medium spiny neurons

**DOI:** 10.1186/1750-1326-8-47

**Published:** 2013-12-26

**Authors:** Ellen M Unterwald, Michelle E Page, Timothy B Brown, Jonathan S Miller, Marta Ruiz, Karen A Pescatore, Baoji Xu, Louis French Reichardt, Joel Beverley, Bin Tang, Heinz Steiner, Elizabeth A Thomas, Michelle E Ehrlich

**Affiliations:** 1 Department of Pharmacology and Center for Substance Abuse Research, Temple University School of Medicine, Philadelphia, PA 19140, USA; 2 Farber Institute for Neurosciences, Thomas Jefferson University, Philadelphia, PA 19107, USA; 3 Departments of Neurology and Pediatrics, Icahn School of Medicine at Mount Sinai, Annenberg 14-44, Box 1137, 1 Gustave L. Levy Place, New York, New York 10019, USA; 4 Department of Pharmacology, Georgetown University Medical Center, Washington, DC 20057, USA; 5 Department of Physiology, University of California, San Francisco, CA 94158, USA; 6 Department of Cellular and Molecular Pharmacology, The Chicago Medical School, Rosalind Franklin University of Medicine and Science, North Chicago, IL, USA; 7 Department of Molecular Biology, The Scripps Research Institute, La Jolla, CA 92037, USA

**Keywords:** TrkB.FL, Medium spiny neuron, Dorsal striatum, BDNF, DARPP-32

## Abstract

**Background:**

The high affinity tyrosine kinase receptor, TrkB, is the primary receptor for brain derived neurotrophic factor (BDNF) and plays an important role in development, maintenance and plasticity of the striatal output medium size spiny neuron. The striatal BDNF/TrkB system is thereby implicated in many physiologic and pathophysiologic processes, the latter including mood disorders, addiction, and Huntington’s disease. We crossed a mouse harboring a transgene directing cre-recombinase expression primarily to postnatal, dorsal striatal medium spiny neurons, to a mouse containing a floxed *TrkB* allele (fB) mouse designed for deletion of TrkB to determine its role in the adult striatum.

**Results:**

We found that there were sexually dimorphic alterations in behaviors in response to stressful situations and drugs of abuse. Significant sex and/or genotype differences were found in the forced swim test of depression-like behaviors, anxiety-like behaviors on the elevated plus maze, and cocaine conditioned reward. Microarray analysis of dorsal striatum revealed significant dysregulation in individual and groups of genes that may contribute to the observed behavioral responses and in some cases, represent previously unidentified downstream targets of TrkB.

**Conclusions:**

The data point to a set of behaviors and changes in gene expression following postnatal deletion of TrkB in the dorsal striatum distinct from those in other brain regions.

## 

Brain-derived neurotrophic factor (BDNF) and its high affinity tyrosine kinase receptor, TrkB, play important roles in development, maintenance and plasticity of the striatal output medium size spiny neuron (MSN). Eighty to ninety percent of the BDNF in the striatum is anterogradely transported from the cortex, and the remainder is derived from the substantia nigra [1]. All MSNs express TrkB, the primary receptor for BDNF, but TrkB is enriched in dopamine D2-receptor (D2R)-expressing MSNs relative to D1R-expressing MSNs [2-4]. TrkB is encoded by the gene, Neurotrophic tyrosine kinase, receptor, type 2 (*Ntrk2*). There are two major TrkB isoforms, including the full-length TrkB.FL containing the tyrosine kinase domain, i.e. tk+, and a C-terminal truncated form, TrkB.T1, lacking the catalytic tyrosine kinase domain. TrkB.T1 is a dominant-negative receptor that inhibits TrkB.FL signaling, but has many other functions, including as a signal transducing molecule [5].

The striatal BDNF/TrkB system is implicated in physiologic and pathophysiologic processes, e.g. mood disorders and addiction, which present as primary diseases and as disorders associated with neurodegenerative diseases, including Alzheimer’s, Huntington’s and Parkinson’s disease. In addition, the system is directly implicated in striatal dysfunction and degeneration in Huntington’s disease. Thus, the BDNF/TrkB system is a potential therapeutic target in a myriad of clinical conditions. An emerging aspect of the study of this system is the dispte, and sometimes opposite, effects on behavior derived from its manipulation in different brain regions, e.g. hippocampus vs. ventral striatum [6]. Within the striatum, studies of mood disorders and addiction focus largely on the ventral portion, but evidence continues to accumulate supporting a role for the dorsal striatum in these pathologies. The dorsal striatum, including the caudate and putamen sub-regions, is an intrinsic part of the circuitry of mood disorders [7], and dorsal striatal dysfunction is particularly relevant in compulsive drug seeking [8] and gating of alcohol intake [9]. The dorsal striatum also obviously plays a key role in Huntington’s and Parkinson’s disease.

We sought to examine the role of the TrkB receptor in mature MSNs in adult male and female mice, with a focus on behavior and gene expression. Previous studies following constitutive and conditional deletion of the TrkB receptor in all MSNs or subtypes [2,4,10,11] were based largely on deletion during the prenatal period. In addition, in the prior studies, TrkB deletion was not restricted to MSNs. A limited number of studies utilizing viral-mediated Cre recombinase, overexpression of TrkB, or siRNA knockdown of TrkB in adult rodents have been published [12,13], but none has compared males to females. To this end, we used a mouse in which Cre-recombinase expression is delayed until the postnatal period, and in which within the forebrain, the transgene is restricted to MSNs [14]. We found sexually dimorphic changes in animal behaviors that are reflective of pathophysiology associated with human mood disorders and drug abuse. We also used a genome-wide assay of the TrkB-deleted dorsal striatal transcriptome to identify downstream TrkB targets which may be associated with alterations in behavior and to compare these alterations to those that occur following prenatal deletion of cortical BDNF.

## Results

### Creation of D9ΔTrkB mouse and assays of TrkB level

**Figure 1:**
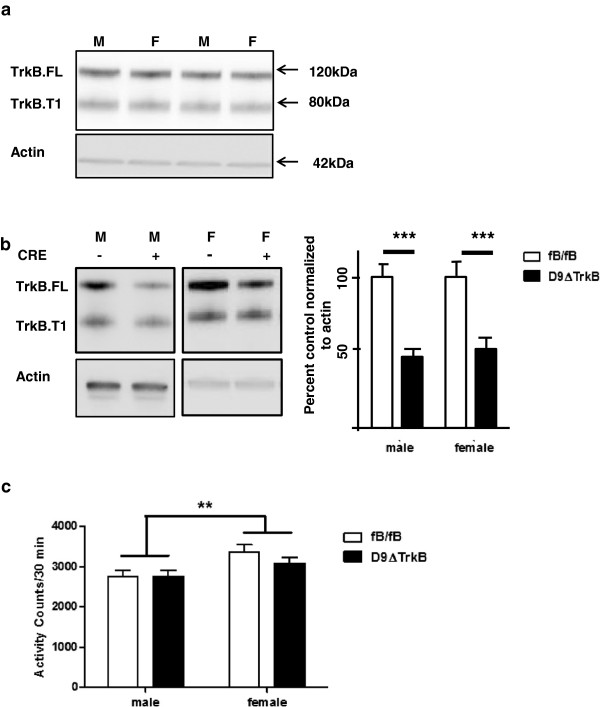
**Baseline striatal TrkB level and locomotor activity in D9ΔTrkB mice. (a)** Baseline levels of striatal TrkB.FL and TrkB.T1 are equal in fB/fB male and female mice. Densitometric values were normalized to actin. Blot is representative of N = 6, p > 0.05 (t-test). **(b)** Relative to fB/fB, D9ΔTrkB male and female mice demonstrate approximately a 50% decrease in striatal TrkB.FL (N = 3–4,*** P < 0.005, t-test). There is a trend towards a small decrease in TrkB.T1 (N = 3, P = 0.06, t-test). Densitometric values were normalized to actin, and fB/fB levels were arbitrarily set to equal 1. **(c)** Down-regulation of striatal MSN TrkB.FL does not alter baseline total activity, but there is a genotype-independent increase in activity in female mice relative to males (N = 8-12/group, ** P < 0.01 two-way ANOVA for sex factor).

We previously created a transgenic mouse (aka D9-Cre) using 9Kb of the *Ppp1r1b* gene to drive expression of Cre-recombinase in postnatal striatal medium spiny neurons [14]. D9-Cre was crossed with a floxed *TrkB* mouse (aka fB), which deletes the signal peptide of TrkB. Importantly, the fB/fB homozygous mouse has a normal level of TrkB [2]. Homozygote fB, heterozygote D9-Cre mice were used for this study, and we refer to them as D9ΔTrkB. Controls were fB/fB only without any expression of cre recombinase. On western blotting of protein from the dorsal striatum, male and female fB/fB mice had equal levels of TrkB.FL (Figure 1a, P > 0.05, not significant). Using western blotting, RT-qPCR and *in situ* hybridization with full-length TrkB-specific primers, we found that TrkB mRNA and protein were decreased by 50% in the dorsal striatum in males and females, and by 25-40% in the ventral striatum/nucleus accumbens in D9ΔTrkB mice compared to fB/fB controls. These data are consistent with our previous report showing that D9-Cre directs recombination in the majority of the dorsal MSNs, with a lower level of recombination in the ventral striatum [14]. The decrease in TrkB.T1 protein did not reach significance in D9ΔtrkB mice (Figure 1b). Of note, striatal interneurons express a higher level of TrkB than is found in MSNs, and glia express a high level of TrkB.T1 [3,5,15]. The D9-Cre transgene is not expressed in either interneurons or glia, and hence the remaining TrkB likely results from expression in these cell types, in addition to pre-synaptic TrkB receptors on glutamatergic terminals. Previous reports with early conditional deletion of TrkB using *Dlx5/6*-Cre recombinase reported an almost complete deletion of both FL and T1 TrkB [2,4], but the percent decreases using more selective Cre drivers or viral-mediated-Cre recombination were similar to that which we observed [10,12].

### D9ΔTrkB male and female mice have normal baseline motor behavior

Each behavior assay was performed on a naïve cohort. Pan-neuronal, prenatal deletion of TrkB usually leads to early postnatal death, but the mice that survive for several weeks display circling, hind limb clasping, dystonic-like features, and ataxia. Selective early, prenatal striatal deletion leads to a failure of MSN development, and is also associated with hind-limb clasping and hypoactivity [2,4]. Life span and adult weight of D9ΔTrkB mice were normal and the mice were free of spontaneous motor abnormalities and hind limb clasping up to 24 months of age. Although females of both genotypes had higher activity counts than males, there were no genotype-dependent differences in total activity, ambulatory activity or stereotypy in 6-month-old male and female D9ΔTrkB and fB/fB mice (Figure 1c). In addition, latency to fall on rotarod testing was unchanged in adult D9ΔTrkB mice indicating no effect of genotype on motor coordination (data not shown).

### D9ΔTrkB male and female mice display genotype- and sex-dependent differences in depression- and anxiety-like behaviors

**Figure 2:**
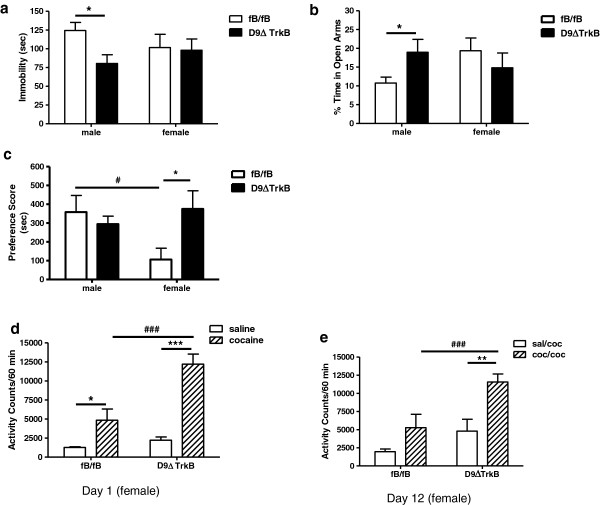
**D9ΔTrkB male and female mice display genotype- and sex-dependent differences in depression- and anxiety-like behaviors and in cocaine place preference. (a)** In the forced swim test, male D9ΔTrkB mice had lower immobility scores versus male fB/fB, but there was no difference for females (*P < 0.05 fB/fB vs D9ΔTrkB males, N = 10-18/group). **(b)** In the elevated plus maze, male D9ΔTrkB spent a greater amount of time in the open arms versus male fB/fB, but there was no difference for females (*P < 0.05 fB/fB vs D9ΔTrkB males, N = 8-10/group). **(c)** Cocaine produced a larger place preference in D9ΔTrkB females versus fB/fB females (*P < 0.05, N = 7/group). Males showed a place preference for cocaine that was genotype-independent. fB/fB males showed a significantly greater place preference than fB/fB females (#P < 0.05, N = 7-8/group). **(d)** Acute cocaine, 20 mg/kg, increased activity in females of both genotypes (*p < 0.05, ***P < 0.001 saline versus cocaine, N = 5-6/group). Cocaine produced greater hyperactivity in female D9ΔTrkB mice versus fB/fB (### P < 0.001, N = 5-6/group). **(e)** Female mice were injected with saline or cocaine, 20 mg/kg, for 5 days. After a 6-day drug-free period, a cocaine challenge injection, 15 mg/kg, produced greater hyperactivity in D9ΔTrkB mice pretreated with cocaine versus fB/fB controls pretreated with cocaine (###P < 0.001, N = 5-6/group) In addition, D9ΔTrkB mice pretreated with cocaine showed greater hyperactivity in response to the cocaine challenge than did D9ΔTrkB mice pretreated with saline (**P < 0.01, N = 5–6), demonstrating that locomotor sensitization was greater in the D9ΔTrkB female mice.

Depressive- and anxiety-like behaviors were assayed in naive cohorts of 6-month-old male and female mice. The forced swim test was used to assess depression-like behavior [16]. Mice were placed individually into a cylinder of water for 6 minutes during which time activity was measured in terms of passive immobility or active swimming and climbing. In this test, increasing periods of immobility are associated with a depression-like phenotype, whereas clinically useful antidepressants reduce immobility [16]. Results of the forced swim test demonstrated that male D9ΔTrkB mice had lower immobility scores versus male fB/fB. One-way ANOVA revealed a significant effect of genotype for the males (F[1,26] = 8.52; P < 0.01; Figure 2a), indicating an anti-depressive phenotype, but no difference for females.

To determine the effects of deletion of striatal MSN TrkB on anxiety, mice were tested in the elevated plus maze [17]. Two-way ANOVA revealed a significant sex by genotype interaction (F(1,29) = 4.279, P < 0.05; Figure 2b). D9ΔTrkB males displayed lower anxiety-like behavior on the elevated plus maze as shown by significantly greater time spent in the open arms compared with control fB/fB male mice (P < 0.05; Figure 2b;). D9ΔTrkB females did not show this effect as there was no significant difference between genotypes in the time spent in the open arms of the elevated plus maze for the females (P > 0.50).

### Cocaine conditioned reward

Conditioned place preference was used to measure the rewarding effects of cocaine (10 mg/kg, ip) in D9ΔTrkB versus fB/fB adult male and female mice. Two-way ANOVA revealed a significant sex by genotype interaction (F(1,26) = 4.935, P < 0.05; Figure 2c). Male mice showed a significant place preference for the cocaine-paired environment with no significant differences between genotypes. Females, however, showed a significant effect of genotype. Cocaine produced a larger place preference in D9ΔTrkB females than in fB/fB female mice (P < 0.05). Further, fB/fB males showed a greater preference for the cocaine-paired environment than fB/fB females (P < 0.05). These results suggest an increase in cocaine reward upon TrkB deletion in the striatum selectively in female mice.

### Locomotor responses to acute and repeated administration of cocaine in female fB/fB and D9ΔTrkB mice

Based on genotype- and sex-specific results in the cocaine place preference assay, we investigated the response to acute and repeated administration of cocaine in female mice to analyze acute activity and stereotypy responses to cocaine, and to determine if D9ΔTrkB female mice sensitized to the motor-stimulating effects of repeated administration of cocaine. Figure 2d shows total activity counts of female D9ΔTrkB and fB/fB mice following the first saline or cocaine (20 mg/kg, ip) injection. Two-way ANOVA revealed a significant effect of genotype (F(1,18) = 16.96, P = 0.0006), treatment (F(1,18) = 45.03, P < 0.0001), and interaction (F(1,18) = 10.06, P = 0.0053). Cocaine produced greater hyperactivity in female D9ΔTrkB mice as compared with female fB/fB controls (P < 0.001). When activity was septed into ambulatory and stereotypy responses, D9ΔTrkB female mice showed higher levels of both ambulatory and stereotypic activity following a cocaine injection (P < 0.01 and P < 0.001, respectively; data not shown).

Next, female mice were injected with saline or cocaine, 20 m/kg ip, once daily for 5 days, which was followed by a 6-day drug-free period. On day 12, all mice were challenged with a cocaine injection (15 mg/kg ip) and activity recorded to test for the development of sensitization. Two-way ANOVA showed a significant effect of genotype (F(1,18) = 12.68, P = 0.0022) and treatment (F(1,18) = 15.42, P = 0.001) for total activity (Figure 2e), D9ΔTrkB female mice, but not control fB/fB females, showed behavioral sensitization as evidenced by significantly greater hyperactivity following the cocaine challenge on Day 12 in D9ΔTrkB pretreated with cocaine than in D9ΔTrkB pretreated with saline (P < 0.01). In addition, a genotype effect was found in that D9ΔTrkB mice pretreated with cocaine showed greater hyperactivity in response to the cocaine challenge as compared with control mice pretreated with cocaine (P < 0.001). There were significant genotype effects for both ambulatory (P < 0.05) and stereotypic activity (P < 0.01) with D9ΔTrkB mice being the high responders in both cases (data not shown). These results demonstrate a hyperactive phenotype of the D9ΔTrkB female mice in response to acute and repeated cocaine administration.

### Alterations in gene expression in dorsal striatum in D9ΔTrkB mice

**Figure 3:**
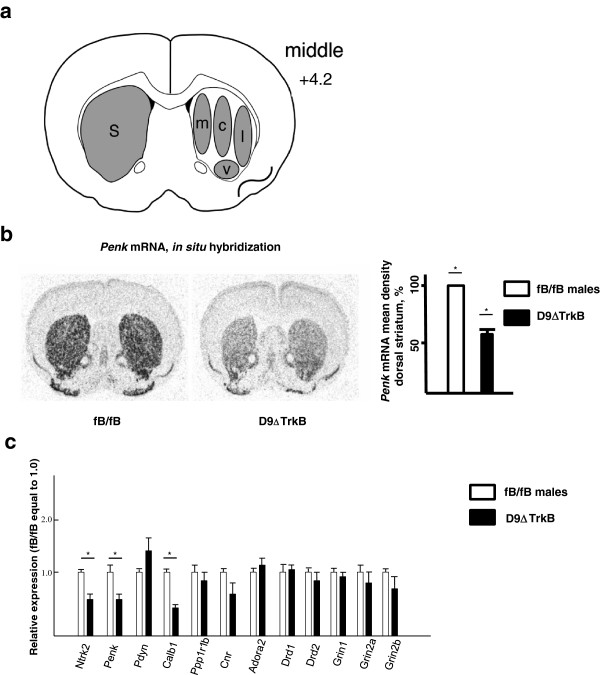
**Down-regulation of striatal MSN TrkB leads to specific alterations in expression of MSN-enriched transcripts in male mice. (a)** Diagram of dorsal striatal regions (middle level, 4.2 mm rostral to the interaural line) used for quantitation of results of *in situ* hybridization of *Ntrk2*, *Penk*, *Tacr1*, *Ppp1r1b* and *Pdyn* mRNAs. S = striatum, m = medial, c = central, l = lateral, v = ventral. **(b)** Densitometry of representative autoradiograms of *Penk* mRNA *in situ* hybridization on mid-level striatal sections shows a 40% reduction in preproenkephalin mRNA level (N = 3 fB/fB, N = 4 D9ΔTrkB, P < 0.01, t-test). **(c)** RT-qPCR was performed on samples derived from the dorsal striatum of male mice (N = 4/genotype), confirming a significant decrease in *Ntrk2* and *Penk* mRNAs in D9ΔTrkB mice versus fB/fB mice (*P < 0.01, t-test) and demonstrating greater than 50% decrease in *Calb1* mRNA (*P < 0.01, t-test).

Prenatal, conditional deletion of cortical BDNF using *Emx1*-cre mediated recombination results in a myriad of changes in the transcriptome [18] and changes in expression of selected genes have been identified following prenatal deletion of TrkB throughout the striatum or in specific MSN subtypes [2,4,10]. Genome-wide assay of the transcriptome following deletion of striatal TrkB at any age or in specific MSN subtypes has not previously been reported, and we reasoned that chronic down-regulation of TrkB in the mature MSN would alter the transcription of multiple genes. *In situ* hybridization of *Ntrk2*, *Penk*, *Tacr1*, *Ppp1r1b* and *Pdyn* mRNAs demonstrated distinct patterns of regulation in male D9ΔTrkB mice and the data are summarized in Table 1. Figure 3a schematically shows dorsal striatal sub-regions that were analyzed at the mid-level of the striatum, the level at which all effects were maximal. In the sub-regions, *Ntrk2* mRNA was decreased from 40-60%, with an overall decrease of 51% (fB/fB 19.7+/−1,7 vs D9ΔTrkB 10.0+/−1.2, arbitrary units, P = 0.005). *Penk* mRNA was decreased from 30-45% with an overall decrease of 38% (Figure 3b; fB/fB 70.0+/−4.1 vs D9ΔTrkB 43.6+/−1.3, P < 0.001). *Ppp1r1b* were decreased in the dorsal striatum, with a medial-lateral gradient, maximal in the lateral region of the middle level (fB/fB 44.0+/−0.5 vs D9ΔTrkB 37.6+/−1.0, P = 0.004). In the same region, *Tacr1* was decreased by 20% (fB/fB 61.7+/−3.7 vs. D9ΔTrkB 50.0+/−2.4, P = 0.04). *Pdyn* was increased in D9ΔTrkB relative to fB/fB, significant only on the middle level (fB/fB 19.2+/−1.1 vs D9ΔTrkB 26.1+/−2.1; P < 0.05). In the nucleus accumbens at +5.2 mm rostral to the interaural line, *Ntrk2* was decreased by 40% (fB/fB 26.2+/−3.6 vs D9ΔTrkB 15.4+/−1.0, P = 0.02). A lower level of Cre-mediated recombination in the nucleus accumbens was most obvious when assaying downstream targets of TrkB deletion, as *Penk* was decreased by only 18% (fB/fB 75.6+/−3.8 vs D9ΔTrkB 61.7+/−2.5, P = 0.02), and *Tacr1*, *Ppp1r1b* and *Pdyn* were unaltered in the nucleus accumbens.

**Table 1:** **Summary of gene expression changes as determined by *in situ *** **hybridization (percent change in D9ΔTrkB relative to fB/fB)**

**Gene ID**	**Dorsal striatum**	**Ventral striatum**
**(mRNA)**	**(caudate-putamen)**	**(nucleus accumbens)**
*Ntrk2*	(−) 51%, P < 0.01	(−) 40%, P < 0.05
*Penk*	(−) 38%, P < 0.001	(−) 18%, P < 0.05
*Ppp1r1b* (lateral)	(−) 15%, P < 0.01	No change
*Tacr1* (lateral)	(−) 20%, P < 0.05	No change
*Pdyn*	(+) 30%,P < 0.05	No change

RT-qPCR assays were then performed with samples derived from the dorsal striatum of male mice (Figure 3c). Since the entire dorsal striatum was dissected for this analysis, it is important to note that sub-region-specific alterations within the striatum were possibly masked. For example, although P*pp1r1b*/DARPP-32 is markedly up-regulated by BDNF during development [19] and was decreased in D9ΔTrkB dorsolateral striatum as measured by *in situ* hybridization, it was unchanged as measured by RT-qPCR. Given that TrkB is enriched in dopamine receptor type 2 (D2R) MSNs relative to dopamine receptor type 1 (D1R) MSNs [2], we performed a screen of transcripts that distinguish between MSN subtypes, e.g. direct pathway D1R neurons, indirect pathway D2R neurons, and markers of patch and matrix MSNs (Figure 3c). We found that levels of *Drd1* and *Drd2* mRNAs were unchanged in dorsal striatum of male D9ΔTrkB mice. The *A2ar*/adenosine receptor type 2 mRNA, which co-localizes with D2R and not with D1R, and subunits of the NMDA receptor were also unchanged. Although morphometric studies were not the focus of this study, the D9ΔTrkB striatum appeared smaller and the lateral ventricles larger than in fB/fB control mice. Normal *A2ar* mRNA level suggests that death of D2R neurons is not excessive, but as BDNF is required for postnatal growth of MSN dendritic complexity and spine density [20], such deficits could contribute both to a decrease in striatal size and striatal dysfunction. Further work is required to determine whether this apparent size difference is significant, and if so, its origin. Consistent with the *in situ* hybridization data, *Penk*, also enriched in D2R indirect pathway MSNs, was markedly decreased, as was *Calb1*/calbindin, which is enriched in the matrix compartment. Also consistent with the *in situ* hybridization, *Pdyn*, enriched in D1R MSNs and the striosome/patch compartment [21], strongly trended upwards (P = 0.07). *Cnr1*, or cannabinoid receptor 1, is present in both D1R and D2R neurons and strongly trended downwards (P = 0.07). These data suggest that genes expressed in MSNs and regulated by TrkB are not relegated to a specific MSN subtype.

**Figure 4:**
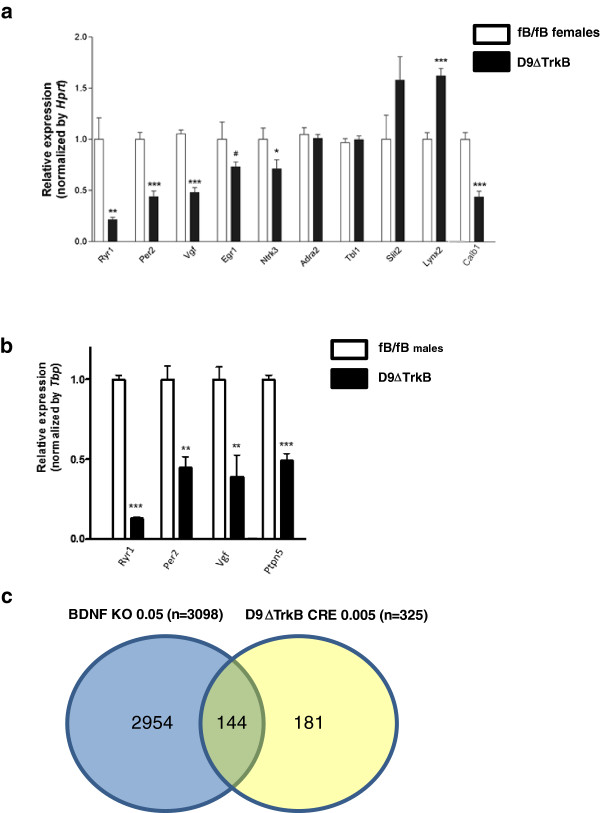
**Microarray analysis of dorsal striatal samples from female D9ΔTrkB mice reveals widespread alterations in gene expression (see Table **2**). (a)** RT-qPCR assay of *Ryr1*, *Slit2*, *Egr1*, *Ntrk3*, *Vgf*, *Per2*, and *Lynx2* mRNAs validated the results of the microarray analysis, whereas assay of *Adra2* mRNA did not show a difference between genotypes (N = 4/genotype; * P < 0.05,**P < 0.01,***P < 0.001). **(b)** RT-qPCR assay of *Ryr1*, *Per2*, *Ptpn5* and *Vgf* mRNAs in striatal samples from male mice (N = 4/genotype) showed reductions in transcripts similar to those observed in samples from females (**P < 0.01,***P < 0.001). **(c)** Venn diagram illustrating that of the top 325 genes with mRNA levels altered at the P < 0.005 level in D9ΔTrkB mice, only 144 were altered in BDNF-null mice (from [18]) at the least stringent level of significance, P < 0.05.

We next performed a microarray analysis of baseline gene expression in female dorsal striatum of fB/fB and D9ΔTrkB mice (N = 4/genotype). There were 1749 transcripts that were altered at the P < 0.05 level of significance (Additional file 1: Table S1). Importantly, there was only one discrepancy for the transcripts assayed in Figure 3c, as *Calb1* mRNA was unchanged by microarray. *Calb1* mRNA was, however, decreased in female D9ΔTrkB striatum by over 50% when assayed by RT-qPCR (Figure 4a). We focused on the 325 transcripts dysregulated in D9ΔTrkB mice at P value less than 0.005 (Additional file 1: Table S1), in order to identify candidate genes potentially relevant to behavioral alterations described herein. Of these, 93 were up-regulated following MSN TrkB deletion, and 260 were down-regulated. Included amongst those that we validated in female mice by RT-qPCR are *Vgf*, *Egr1*, *Lynx2*, *Per2*, *Ryr1* and *Slit2* (Figure 4a), chosen in large part for their known or hypothesized associations with behaviors assayed in this study and/or synaptic plasticity. Of note, several of these have not previously been associated with the BDNF/TrkB signal transduction system, e.g*. Per2. Ntrk3/*TrkC was significantly down-regulated, indicating that not only does this neurotrophin receptor not compensate for the knockdown of *Ntrk2*/TrkB, but that it is a direct or indirect downstream target of *Ntrk2* and may contribute to the alterations following deletion of MSN TrkB.

We interrogated the microarray data to identify pathways and functional classes over-represented in the list of altered transcripts. Using GOEAST and DAVID at both P < 0.005 and 0.01 levels of significance, enrichment and gene clusters were found in the following Gene Ontology (GO) classifications: metal ion transport, voltage gated ion channel activity, and metal ion and cation membrane transporter activity. These pathways and molecules are associated with intracellular calcium regulation and signal transduction, and some are known to be required for BNDF activity [22]. Amongst them is the aforementioned *Calb1* and *Ryr1*, and also *Calb2*, *Hpcal4*, *Cacng5*, and *Homer1*, the latter known to be associated with BDNF activity and plasticity [23].

We next used Ingenuity Systems Pathways Analysis to examine canonical pathways that were significantly correlated with genes differentially expressed in the striatum of D9ΔTrkb mice. Top pathways included Protein kinase A signaling, G-protein signaling, CXCR4 signaling and RAR activation (Table 2). The Protein kinase A signaling pathway contained *Ptpn5*/STEP, a striatal-enriched gene known to be associated with responses to drugs of abuse and stress [24], and not previously identified as being regulated by BDNF/TrkB. In addition, there were alterations in several genes encoding members of the cAMP-mediated signaling pathway, e.g. *Rgs2* and *Adcy1*, and other signal transduction pathways, including CREB and ErbB2-ErbB3 (Table 2).

**Table 2:** Top canonical pathways associated with TrkB deletion in mouse striatal MSNs

**Canonical pathway:**	**-log (p-value):**	**Ratio:**	**Molecules:**
Thrombin signaling	2.69E + 00	5.34E-02	ARHGEF10, MPRIP, GATA1, RHOT2, ADCY1, GNB5, CAMK1G, HRAS, PRKCH, GNG7, PRKCB
Protein kinase A signaling	2.63E + 00	4.28E-02	SHH, PTPN7, PTPRE, SMAD3, DUSP6, GNB5, PTPN5, TCF7, GNG7, PTEN, ADD3, ADCY1, SMAD4, RYR1, PRKCH, CDC16, PRKCB
Galpha(i) signaling	2.44E + 00	6.06E-02	GRM8, NPY1R, ADCY1, GNB5, HRAS, ADRA2C, STAT3, GNG7
Prolactin signaling	2.39E + 00	7.50E-02	HRAS, PRKCH, STAT3, SOCS5, TCF7, PRKCB
CXCR4 signaling	2.36E + 00	5.36E-02	EGR1, RHOT2, ADCY1, GNB5, HRAS, PRKCH, ELMO1, GNG7, PRKCB
Glutamate receptor signaling	2.18E + 00	7.25E-02	GRM8, SLC1A1, HOMER1, GNG7, GRIK1
Galpha(s) signaling	2.11E + 00	5.79E-02	RGS2, ADD3, ADCY1, GNB5, RYR1, HTR6, GNG7
Cell cycle: G1/S checkpoint regulation	2.06E + 00	7.58E-02	PA2G4, HDAC8, SMAD3, SMAD4, CDKN1B
Beta-adrenergic signaling	2.04E + 00	5.71E-02	ADCY1, GNB5, HRAS, PRKCH, GNG7, PRKCB
Phospholipase C signaling	2.04E + 00	4.23E-02	ARHGEF10, MPRIP, HDAC8, RHOT2, ADCY1, GNB5, ITGA5, HRAS, PRKCH, GNG7, PRKCB
Neuregulin signaling	2.00E + 00	5.88E-02	ITGA5, HRAS, PRKCH, CDKN1B, PTEN, PRKCB
G Beta gamma signaling	2.00E + 00	5.13E-02	ADCY1, GNB5, HRAS, PRKCH, GNG7, PRKCB
RAR activation	1.98E + 00	4.76E-02	SMAD3, ADCY1, SMAD4, PRKCH, ERCC2, PTEN, PPARGC1A, RBP4, PRKCB
Virus entry via endocytic pathways	1.97E + 00	6.06E-02	ITGA5, TFRC, HRAS, PRKCH, CXADR, PRKCB
Chronic myeloid leukemia signaling	1.86E + 00	5.71E-02	PA2G4, HDAC8, SMAD3, SMAD4, HRAS, CDKN1B
Glioma signaling	1.84E + 00	5.36E-02	PA2G4, CAMK1G, HRAS, PRKCH, PTEN, PRKCB
Axonal guidance signaling	1.77E + 00	3.42E-02	SHH, GNB5, HRAS, SEMA6B, ITGA5, FZD1, SLIT2, GIT1, GNG7, NTNG1, SEMA3A, SUFU, BAIAP2, ABLIM2, PRKCH, PRKCB
Breast cancer regulation by stathmin1	1.73E + 00	4.35E-02	ARHGEF10, ADCY1, GNB5, CAMK1G, HRAS, PRKCH, CDKN1B, GNG7, PRKCB
Molecular mechanisms of cancer	1.60E + 00	3.44E-02	SHH, PA2G4, SMAD3, RHOT2, HRAS, FZD1, ARHGEF10, SUFU, ADCY1, SMAD4, PRKCH, CDKN1B, PRKCB
Renin-angiotensin signaling	1.60E + 00	4.80E-02	ADCY1, HRAS, SHC3, PRKCH, STAT3, PRKCB
CREB signaling in neurons	1.56E + 00	3.94E-02	GRM8, ADCY1, GNB5, HRAS, PRKCH, GNG7, GRIK1, PRKCB
Androgen signaling	1.55E + 00	4.17E-02	SMAD3, GNB5, PRKCH, ERCC2, GNG7, PRKCB
ErbB2-ErbB3 signaling	1.51E + 00	6.67E-02	HRAS, STAT3, CDKN1B, PTEN
Calcium signaling	1.48E + 00	3.79E-02	TNNT1, HDAC8, CAMK1G, TPM2, RYR1, TRPC4, CASQ2, GRIK1
14-3-3-mediated signaling	1.45E + 00	4.96E-02	STK11, TSC2, HRAS, PRKCH, CDKN1B, PRKCB
P2Y purigenic receptor signaling pathway	1.42E + 00	4.35E-02	ADCY1, GNB5, HRAS, PRKCH, GNG7, PRKCB
PTEN signaling	1.42E + 00	4.44E-02	INPP5F, FLT1, ITGA5, HRAS, CDKN1B, PTEN
IL-8 signaling	1.41E + 00	3.90E-02	FLT1, RHOT2, GNB5, HRAS, PRKCH, IRAK3, GNG7, PRKCB
cAMP-mediated signaling	1.41E + 00	4.02E-02	RGS2, GRM8, NPY1R, DUSP6, ADCY1, CAMK1G, ADRA2C, HTR6, STAT3

Microarray data were analyzed through the use of IPA (Ingenuity® Systems, http://www.ingenuity.com).

The p-value was calculated using the right-tailed Fisher Exact Test, considering the number of molecules participating in a given pathway and the total number of molecules associated with that pathway in Ingenuity’s knowledge base.

Transcripts for multiple immediate early genes and other transcription factors or co-regulators were found to be significantly altered in D9ΔTrkB mice, implying that the dysregulated genes are likely comprised of direct and indirect targets of TrkB signaling. These include several immediate early genes and transcription factors associated with plasticity and responses to drugs of abuse, e.g. *Arc*, *Egr1*, *2* and *4*, and known to be up-regulated by BDNF and/or required for its downstream gene regulation [25,26]. Others include *Dusp6*, *Pou3f1*, *Klf5*, *Calcoco1*, and *Ppargc1α.* Interestingly, *Ppargc1α* is up-regulated, contrary to what would be anticipated by studies in Huntington disease models in which cortical BDNF, striatal TrkB, and Ppargc1α are all down-regulated (reviewed in [27]).

An obvious question is whether sexual dimorphism in gene regulation may account for sex-dependent behavioral differences in D9ΔTrkB mice. Although we did not perform a second microarray analysis on male mice, we did assay levels of several potentially relevant transcripts that we had assayed in female mice either by qPCR or as part of the microarray analysis, including *Ptpn5* (striatum-enriched protein tyrosine phosphatase), *Ryr1*(ryanodine receptor 1), *Vgf* (nerve growth factor inducible), and *Per2* (periodic circadian clock 2). All four transcripts were altered in the same direction and magnitude as in the female mice (Figure 4b).

## Discussion

This is the first report to directly address the effects of postnatal knockdown of MSN TrkB throughout the striatum, but with a focus on the dorsal portion, i.e. caudate-putamen. Previous studies utilized mice expressing the *Dlx5/6*-, *Drd1*- and *Drd2*-cre recombinase drivers, and thereby deleted TrkB in the prenatal period in both dorsal and ventral striatum and in cell types other than MSNs. Viral-mediated manipulation of FL or T1 TrkB in the adult mouse has focused on the ventral striatum. By using a promoter that fortuitously directs expression of Cre-recombinase to postnatal MSNs, the mice studied herein are unlikely to have marked developmental deficits and their attendant molecular compensations. We found that 1) D9ΔTrkB mice have normal lifespan and baseline behavior; 2) there is a sexually dimorphic effect on performance in the forced swim test and elevated plus maze, detectable only in D9ΔTrkB males; 3) the response to cocaine in D9ΔTrkB mice is also sexually dimorphic, with an increased genotype-dependent preference for cocaine in females; 4) female D9ΔTrkB mice show increased locomotor sensitization to chronic cocaine relative to fB/fB controls; and 5) there is a marked effect of TrkB down-regulation on baseline gene expression in the dorsal striatum.

Sexual dimorphism in the response to physiological and behavioral stress, and the direct relationship of this response to the development of mental illness, may be organizational and/or activational (reviewed in [28,29]. The caudate-putamen is a sexually dimorphic brain region, based on sex-specific differences in size and in the number of gonadal hormone receptors [30]. In our study, female mice were group housed, with D9ΔTrkB and fB/fB mice in the same cages. Female mice were not ovariectomized, and estrus cycle was not determined prior to or after testing. It is highly unlikely therefore, that the observed sexual dimorphism was dependent on circulating sex steroid, suggesting organizational effects independent of circulating hormone levels. This hypothesis is being tested in ongoing experiments. Our study contributes to the emerging literature on sexual dimorphism in the neurotrophic system. There are interactions between sex steroid hormones and BDNF in many species, organs, and brain regions, which may modulate transcription, translation, and/or strength of signal transduction of BDNF and TrkB [31-35]. Our data imply that adult males may be more sensitive to a decrease in striatal TrkB level, but perhaps surprisingly, the changes were not detrimental per se, and may even be considered beneficial. Data from studies of whole brain and/or hippocampus [36-38] largely show that a decrease in the level of BDNF, and by extension TrkB, generally leads to an increase in depressive- and anxiety-related behaviors or in females, a lower threshold for stress-induced anhedonic behavior. An important exception in the literature is a strong trend to a decrease in anxiety-related behavior in female mice following forebrain deletion of BDNF [37]. On the other hand, BDNF in the nucleus accumbens is pro-depressant and an experimental decrease in BDNF or an increase in TrkB.T1 in the nucleus accumbens is “anti-depressant” in male mice [6]. A decrease in depressive- and anxiety-related behaviors in male mice following striatal TrkB knockdown is also consistent with a recent report of similar effects following administration of a TrkB antagonist [39]. Thus, albeit highly speculative, a decrease in TrkB activity in the striatum appears to “override” a decrease in the hippocampus, and knockdown in the dorsal striatum is required for an effect on anxiety-related behaviors, which are not altered by knockdown of the BDNF/TrkB system restricted to the nucleus accumbens, or to D1R or D2R MSNs. It is therefore important to note that in studies utilizing forebrain deletion of BDNF [37,38], the amount of BDNF transported from the ventral tegmental area to the nucleus accumbens may not have been altered. Together, these data encourage further investigation into the role of the dorsal striatum in depression and anxiety, and the apparent differences that arise from whole brain and region-specific manipulation of BDNF or TrkB.

The question of the regional and neuronal subtype origins of behavioral changes is also relevant to the responses to cocaine observed in this study. It remains difficult to parse out exclusive functions for the ventral and dorsal striatum, but the reward mechanism as measured by preference for cocaine in the conditioned place preference pdigm is largely attributed to the ventral striatum whereas acute locomotor activity and locomotor sensitization to cocaine are primarily mediated by the dorsal striatum [40-43]. The dorsal striatum, however, is strongly implicated in reward mechanisms based on habit learning theories of addiction [8], as particularly demonstrated in models of an escalation in cocaine seeking that implicate BDNF signal transduction [44,45]. Deletion of TrkB in dorsal and ventral striatal D1R MSNs results in increased preference for cocaine and increased sensitization in male mice, whereas deletion in D2R MSNs (and presumably some interneurons) has opposite effects [10]. A decrease in accumbal TrkB in both D1R and D2R MSNs reduces cocaine reward [12,13]. Prenatal deletion of TrkB in *Penk*-containing MSNs results in baseline hyperlocomotion and an enhanced locomotor response to cocaine in males, but sensitization and cocaine preference were not assayed in this study [11]. Our data imply that in females, the role of the D1R MSNs overrides that of the D2R MSNs when TrkB is down-regulated in both MSN subtypes during the postnatal period. They may also suggest that accumbal TrkB is a limiting factor in previously reported responses, as we saw a lesser and more variable decrease in TrkB in this region. These data therefore reinforce the relevance of the dorsal striatum and the difficulty in attributing behaviors specifically to the dorsal or ventral regions [42], and highlight the need for increased attention to female models of disease.

We identified many gene expression abnormalities that were associated with TrkB down-regulation in MSNs. Of the 325 genes identified in the present study with transcript levels altered at P < 0.005, only 144 of these transcripts have been reported to be altered in BDNF-null mice at the least stringent level of significance, P < 0.05 [18] (Figure 4c). These data suggest that the MSN TrkB receptor is mediating a distinct subset of behavioral and physiological effects elicited by BDNF in the striatum. In addition, it is possible that prenatal disruption of striatal BDNF/TrkB signaling may produce more widespread alterations of the transcriptome than postnatal down-regulation of BDNF/TrkB signaling, although expression of BDNF in the cortex is low in the prenatal period. Finally, it is possible that the broader disruption of the striatal transcriptome following deletion of BDNF represents other cell types and/or non-cell-autonomous effects on MSN transcription. Although not directly compble, it is interesting to note that of the 20 transcripts with the greatest fold-change in our microarray analysis, only one is altered in the nucleus accumbens following cre-mediated deletion of BDNF in the adult ventral tegmental area [46], and several transcripts with lower fold-change, e.g. *Per2* and *Egr3*, are altered in opposite directions.

It is tempting, but premature, to assign changes in behavior that we measured in the D9ΔTrkB mice to the observed altered gene expression patterns, i.e. metal ion transport or second messenger signaling; however it is possible that chronic, baseline alterations may have different effects than drug-induced alterations. It is also likely that changes in individual genes may antagonize each other, and so effects of transcripts that were studied in isolation may not be as pertinent when they are altered as part of a wider effect on the transcriptome. For example, *Per2* is not a known target of BDNF/TrkB, and an isolated reduction in Per2 is predicted to increase cocaine preference [47], but only females displayed a genotype-dependent difference in cocaine preference. Differences in regional effects are also important. Strikingly, a decrease in Vgf peptide in the hippocampus is strongly associated with pro-depressant effects [48], yet male D9ΔTrkB mice display anti-depressant activity despite a 60% decrease in Vgf in the striatum. It thus also remains to be determined whether alterations in the transcriptome are sexually dimorphic, given that our gene expression comparison between sexes was limited. The striatum displays a higher number of sex-biased genes than do the hippocampus and cortex, including in the expression of *Ntrk3* and *Pdyn*[49,50]. Protein kinase A signal transduction is also altered in a sexually dimorphic manner after self-administration of cocaine, an effect that could be synergistic with chronic changes in expression of proteins whose activity is regulated by phosphorylation, e.g. Ppp1r1b/DARPP-32 [51]. Finally, the list of striatal genes dysregulated following deletion of TrkB includes genes that are expressed at sexually dimorphic levels in other brain regions, but remain to be studied in the striatum, e.g. the aforementioned *Per2*. Thus, the microarray data provide important and novel leads for targeted study of genetic and epigenetic regulation of sexual dimorphism in the striatum, particularly as related to the BDNF/TrkB system.

## Conclusions

In summary, knockdown/deletion of adult striatal, MSN TrkB results in alterations of behavior and gene expression which differ qualitatively and quantitatively from those that occur following prenatal deletion. Changes in behaviors in response to stressful situations or drugs are sexually dimorphic, but it remains to be determined whether the same is true for prenatal deletion and gene expression. The data encourage further investigation into the role of the dorsal striatum and individual TrkB targets in these behaviors.

## Methods

### Genotyping

Mice were group housed, given ad libitum access to food and water, and housed under a 12-hour light/dark cycle. All animal procedures were conducted in accordance with the National Institute of Health Guidelines for the Care and Use of Experimental Animals and were approved by the Institutional Animal Care and Use Committee at the Icahn School of Medicine at Mount Sinai. Mice with a floxed TrkB allele (fB) [2] and the D9-Cre recombinase mouse [14] have been previously described. Both strains were in the C57BL/6 J background. Tail DNA was prepared from three week old mice. To distinguish between floxed TrkB heterozygote and homozygote mice, the sequence of the PCR primers were as follows: Trkb-n2: ATG TCG CCC TGG CTG AAG TG; Trkb-c8: ACT GAC ATC CGT AAG CCA GT; Trkb-c7: GAT GAT TTC TAG CCT TTT CTG G. TrkB-n2 and Trkb-c8 amplify a 369 bp product from the wild type TrkB allele, whereas Trkb-n2 and Trkb-c7 amplify a 245 bp product from the fB allele. Cre recombinase is detected using a second set of sequence-specific primers [14]. D9-Cre/fB was crossed with fB/fB, which has a normal level of TrkB in the homozygous state [2]. We refer to fB/fB mice expressing D9-cre as D9ΔTrkB. Six to ten month old mice were used for this study.

### Western blotting

Protein levels were measured by western blot analysis of protein extracted from regionally dissected brain tissue. Soluble protein was extracted from tissue in 20 mM Hepes (pH 7.6), 150 mM NaCl, 0.5 mM EDTA, and 0.5% Triton X-100, supplemented with 1X Complete protease inhibitor cocktail (Roche Diagnostics GmbH, Mannheim, Germany), 1 mM PMSF, 50 mM NaF, and 1 mM Na-orthovanadate. Protein levels in the extracts were determined using the Bio-Rad DC Protein Assay. Antibodies used included anti-TrkB (1:1000) (BD Biosciences, Sparks, MD, USA) and anti-β-actin (1:1000, Sigma, St. Louis, MO). Blots were developed and visualized using the Fujifilm LAS-4000 Plus Gel Documentation System and Reader (Fujifilm Holdings Corporation, Tokyo, Japan). Densitometric values were obtained using Multi Gauge software for analysis (Fujifilm Lifesciences, Tokyo, Japan).

### Forced Swimming Test (FST)

Mice were subjected to the swim chamber after an acclimation period (2 hours) in the staging area outside of the testing room. Mice were placed individually into a glass cylinder (46 cm tall x 21 cm in diameter) filled with water (23-25°C water) to a depth of 15 cm for six minutes. The depth is deep enough so that mice cannot support themselves by placing their paws on the base of the cylinder. A cylinder of this diameter was used because the larger swimming area has been reported to increase the predictive validity of the mouse FST [52]. The experiment was videotaped from above and scored later by a rater blind to the genotype. The total duration of immobility during the last 4 min of the test was determined from videotapes.

### Elevated plus maze

The elevated plus maze was used to measure anxiety-like behaviors. The mouse elevated plus maze (San Diego Instruments, San Diego, CA) consists of four equal-sized runways (12 inches long and 2 inches wide) laid-out in the shape of a plus sign and elevated off the ground by 15 inches. Two of the arms are enclosed by solid walls, 6 inches high on the long sides (closed arms), whereas the other two arms have no sides (open arms). A ledge (1/8 inch high) is present around the perimeter of the open arms of the maze. Test room lighting was adjusted such that open arm light levels were w200 lux and closed arm light levels were w160 lux. On test day, mice were acclimated to the room for 60 minutes prior to testing. Each mouse was placed on the center maze and their behavior was recorded for 10 minutes. The videotapes were scored at a later time by a person blind to the genotype. The number of entries into the open and closed arms and the time spent in each arm were measured. An entry was counted when the superior portion of the mouse including the head and neck, the shoulders, the forelimbs and forepaws, and the thoracic region moved into an arm.

### Locomotor activity

Activity was measured using the Digiscan D-Micropro automated activity monitoring system (Accuscan, Inc., Columbus, OH). The activity monitors consist of transparent plastic boxes (45x20x20 cm) set inside metal frames that are equipped with 16 infrared light emitters and detectors. The number of photocell beam breaks is recorded by a computer interface in 5 minute bins. Mice were placed into the activity monitors and activity was recorded for 30 minutes during acclimation to the chambers. After 30 minutes, cocaine (20 mg/kg ip) or saline (3 ml/kg ip) was administered and mice were immediately returned to the activity monitors for 60 additional minutes of activity measurement. This was repeated once daily for 5 days. After a 6-day drug-free period, all mice were injected with cocaine (15 mg/kg ip) and activity was measured as before in order to test for behavioral sensitization.

### Conditioned place preference

Conditioned place preference was performed using a two compartment apptus. The two compartments were of equal size but one had solid black walls and a smooth floor whereas the other compartment had black and white vertical striped walls and a rough floor. There was a removable wall between the two compartments of the apptus. Mice were placed in the apptus with free access to both compartments in a 30 minute pretest session. Time in each compartment was measured. Mice were conditioned for 30 minutes once daily for 4 days. On days 1 and 3, mice were injected with cocaine (10 mg/kg ip) and confined to one compartment. On days 2 and 4, mice were injected with saline (3 ml/kg ip) and confined to the other compartment in a counterbalanced design. The test session occurred 24 hours after the last conditioning session and lasted 30 minutes. During the test session, mice were placed into the apptus with the wall between the compartments removed, identical to the pretest, and time spent in each compartment recorded. Preference scores are reported as time in the cocaine-paired compartment after conditioning minus time in the same compartment prior to conditioning in seconds.

### *In situ* hybridization

Briefly [53], the mice were killed with CO_2_, and their brains were rapidly removed and frozen in isopentane cooled on dry ice. Brains were stored at −20°C until cryostat sectioning. Coronal sections (12 μm) were thaw-mounted onto glass slides (Superfrost/Plus; Daigger, Wheeling, IL, USA), dried on a slide warmer and stored at −20°C. Tissue sections were fixed in 4% pformaldehyde/0.9% saline for 10 min at room temperature, incubated in a fresh solution of 0.25% acetic anhydride in 0.1 M triethanolamine/0.9% saline (pH 8.0) for 10 min, dehydrated, defatted for 2X5 min in chloroform, rehydrated, and air-dried. The slides were then stored at −30°C until hybridization. Oligonucleotide probes (48-mers; Life Technologies, Rockville, MD, USA) were labeled with [35S]-dATP. The probes had the following sequence: for *Tacr1*/substance P, complementary to bases 20–67, GenBank accession no. M68909; *Penk*/enkephalin, bases 304–351, M13227; *Pdyn*/dynorphin, bases 807–854, AF026537; *Ntrk2*/TrkB, bases 733–780, NM 008745; *Ppp1r1b*/DARPP-32, bases 1253–1300, BC031129. Striatal gene expression was determined in the nucleus accumbens (total area, at ~5.2 mm rostral to the interaural line [54]; and in the mid-level striatum (total area and medial, central, lateral and ventral sectors; 4.2 mm rostral to the interaural line; Figure 3a). Gene expression was measured by densitometry (NIH Image; Wayne Rasband, NIMH) on film autoradiograms.

### Statistics

Statistical analysis was conducted using GraphPad software (GraphPad Software, La Jolla, CA). In the case of comparison between genotypes, unpaired two-tailed Student t-test was used. One-way ANOVA or two- way ANOVA with sex and genotype or drug and genotype factors were used to analyze the behavioral data. Bonferroni post-hoc comparisons were used with significant ANOVAs. Unpaired t-test was used to analyze western blot, *in situ* hybridization and RT-qPCR data. Results were considered significant when P < 0.05.

### Microarray analysis

Total RNA was extracted from the striatum of fB/fB and D9ΔTrkB transgenic mice using the Nucleospin RNA kit (BD Biosciences, San Jose, CA). RNA quantity was assessed with Nanodrop (Nanodrop Technologies) and quality with the Agilent Bioanalyzer (Agilent Technologies). Total RNA (200 ng) was amplified, biotinylated and hybridized on Illumina Mouse_8_V2 Beadchips, querying the expression of ~22,000 Refseq transcripts, as per manufacturer’s protocol. Slides were scanned using Illumina BeadStation and signal extracted using Illumina BeadStudio software (Illumina, San Diego CA). Raw data was analyzed as previously described using Bioconductor packages. Quality assessment was performed looking at the inter-array Pearson correlation and clustering based on top variant genes was used to assess overall data coherence. Contrast analysis of differential expression was performed using the ‘limma’ package [55]. After linear model fitting, a Bayesian estimate of differential expression was calculated. Data analysis was aimed at identifying transcriptional changes in the D9ΔTrkB compared to fB/fB mice using different uncorrected p-value cut-offs ranging from p < 0.001 to p < 0.05.

### Pathway analyses

Gene Ontology (GO) terms were identified using the Database for Annotation, Visualization and Integrated Discovery (DAVID) (http://david.abcc.ncifcrf.gov/) to understand the potential biological relevance of differentially expressed genes. Canonical pathway analysis was performed using Ingenuity Systems Pathway Analysis (http://www.ingenuity.com).

## Competing interests

The authors declare that there is no conflict of interest.

## Authors’ contributions

EMU, MEP, TBB, EAT, HS and MEE conceived and designed the experiments, performed experiments, reviewed and interpreted primary data, and were involved in drafting and editing the manuscript. BX and LFR contributed reagents and edited the manuscript. JSM, KAP, JB, MR,and BT designed and performed behavior, *in situ* hybridization or molecular genetic experiments, interpreted primary data and approved the final manuscript. All authors read and approved the final manuscript.

## Supplementary Material

Additional file 1: Table S1 Genes differentially expressed in striatum of D9ΔTrkB mice at P < 0.05. Click here for file
